# OpenAI’s Sora in ophthalmology: revolutionary generative AI in eye health

**DOI:** 10.1038/s41433-024-03098-x

**Published:** 2024-04-30

**Authors:** Ethan Waisberg, Joshua Ong, Mouayad Masalkhi, Andrew G. Lee

**Affiliations:** 1 Department of Ophthalmology, University of Cambridge, Cambridge, UK; 2 Department of Ophthalmology and Visual Sciences, University of Michigan Kellogg Eye Center, Ann Arbor, MI USA; 3 School of Medicine, University College Dublin, Dublin, Ireland; 4 Department of Ophthalmology, Blanton Eye Institute, Houston Methodist Hospital, Houston, TX USA; 5 The Houston Methodist Research Institute, Houston Methodist Hospital, Houston, TX USA; 6 Departments of Ophthalmology, Neurology, and Neurosurgery, Weill Cornell Medicine, New York, NY USA; 7 Department of Ophthalmology, University of Texas Medical Branch, Galveston, TX USA; 8 University of Texas MD Anderson Cancer Center, Houston, TX USA; 9 Texas A&M College of Medicine, Bryan, TX USA; 10 Department of Ophthalmology, The University of Iowa Hospitals and Clinics, Iowa City, IA USA

**Keywords:** Health care, Anatomy

## Introduction

Sora is a state-of-the-art AI model developed by Open AI that has been engineered to generate realistic and imaginative scenes purely based on textual instructions [1]. This innovative application is a remarkable leap forward and highlights the advanced capabilities of modern AI in interpreting and visualizing complex narratives [1]. The technological foundation of Sora rests on Large Language Models (LLMs) and artificial video generation techniques. LLMs are advanced neural network architectures designed to understand, generate, and interpret human language in a highly sophisticated manner [2–5]. When combined with diffusion models for video generation, these AI systems can create detailed and dynamic visual content from text descriptions [1]. This involves processing the text to understand its meaning and context, and then translating them into a series of images that form a coherent video sequence.

**Fig. 1:**
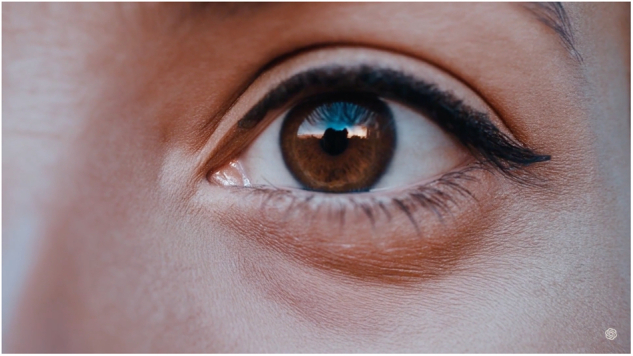
Screencap from video generated by Sora from the prompt: Extreme close-up of a 24-year-old woman’s eye blinking, standing in Marrakech during magic hour, cinematic film shot in 70 mm, depth of field, vivid colors, cinematic. (Open AI).

The implications of such technology extend across various fields, including ophthalmology (Fig. 1). Sora and similar AI models could revolutionize patient education, surgical training, and the visualization of complex eye conditions and visual phenomena. By generating detailed visual simulations based on textual case descriptions or surgical procedures, practitioners can enhance their understanding and teaching of intricate ophthalmic concepts, thereby improving patient care and outcomes. Similarly, it could enable practitioners get an accurate first-person perspective into what their patients are seeing/experiencing, which could provide guidance for improved care and empathy. Despite its ground-breaking potential, at the time of writing, Sora is currently inaccessible for public use and is only available to select individuals.

### Surgical training

Ophthalmic surgery is a highly technical and learning new surgical techniques can be time intensive. Sora can be used to generate step-by-step surgical technique videos from text descriptions. While there are written step-by-step text explanations, with a few photos to describe ophthalmic surgical techniques, an AI-generated surgical video can provide an invaluable visual aid to ophthalmology trainees. A study by Reck-Burneo et al. [6] found that surgical trainees reported having significantly higher levels of confidence following watching operative videos, rather than reading a peer-reviewed manuscript on a surgical technique.

### Patient Education

Strong ophthalmologist-patient communication is essential in the management of eye disease. Helping to educate and empower patients with conditions such as glaucoma, has been shown to improve both clinical outcomes [7] and treatment adherence [8]. A systematic review by Farwana et al. [9] showed that video-based media can be a useful ophthalmic patient education tool, with 71% of studies showing a significant improvement in comprehension following a video intervention. The current standard of providing additional written information, which is usually written in small text on leaflets, is also not particularly well-suited for individuals with visual impairments, non-native English speakers, or individuals with low literacy levels [9].

### Public awareness campaigns

To reduce preventable blindness and vision impairments, the general public must be aware of the importance of regular eye examinations. This was also a key recommendation made by the World Health Organization’s World Report on Vision [10], to empower people and improve eye health literacy worldwide as early detection and timely management can help reduce preventable visual impairments. Ophthalmologists can use Sora to rapidly generate high-quality public awareness campaigns to educate the general public about various ophthalmic disorders and preventative measures that can be taken.

### Clinician education

A video generated by Sora can potentially illustrate symptoms and signs of rare/uncommon ophthalmic diseases, to help improve the ability of ophthalmologists-in-training to recognize them. Ophthalmology residents could then observe and diagnose these conditions in a supportive and controlled environment.

It is also important to also consider possible limitations of Sora. Like all LLMs, minor misunderstandings in written text can lead to the production of inaccurate videos [11–14]. Future research will also need to be conducted on the anatomical accuracy of the ophthalmic AI-generated content.

Other future directions of Sora should include providing audio descriptions of videos to improve accessibility of the content for individuals with vision impairments. All things considered, Sora’s artificial video generation has the potential to enhance ophthalmic surgical training, improve patient education, and the visualization of complex eye conditions and visual phenomena.
